# Intrinsic velocity differences between larynx raising and larynx lowering

**DOI:** 10.1371/journal.pone.0281877

**Published:** 2023-02-16

**Authors:** Christian Kleiner, Patrick Häsner, Peter Birkholz

**Affiliations:** Institute of Acoustics and Speech Communication, Faculty of Electrical and Computer Engineering, Technische Universität Dresden, Dresden, Germany; Chonnam National University Medical School, KOREA, REPUBLIC OF

## Abstract

In this study, 23 subjects produced cyclic transitions between rounded vowels and unrounded vowels as in /o-i-o-i-o-…/ at two specific speaking rates. Rounded vowels are typically produced with a lower larynx position than unrounded vowels. This contrast in vertical larynx position was further amplified by producing the unrounded vowels with a higher pitch than the rounded vowels. The vertical larynx movements of each subject were measured by means of object tracking in laryngeal ultrasound videos. The results indicate that larynx lowering was on average 26% faster than larynx raising, and that this velocity difference was more pronounced in woman than in men. Possible reasons for this are discussed with a focus on specific biomechanical properties. The results can help to interpret vertical larynx movements with regard to underlying neural control and aerodynamic conditions, and to improve movement models for articulatory speech synthesis.

## Introduction

Many studies aim for a better understanding of speech production through the analysis of kinematic quantities, such as the velocity of an articulator when it approaches the target for a certain speech sound [1–5]. This velocity is actively controlled by the nervous system, and it further depends on properties of the biomechanical system and on aerodynamic conditions [6]. The individual contributions of these three factors to an observed articulatory movement are of great interest in speech production research, but they are usually hard to disentangle. When one articulator moves on average slower or faster than another one across different phonetic contexts and different aerodynamic conditions, this articulator-dependent velocity difference is likely due to different biomechanical properties of the articulators. For example, the movement of the tongue towards and away from a velar closure is generally slower than that of the lips during the formation or the release of a bilabial closure [7], which is likely related to the different masses of the tongue and the lips. Furthermore, there is evidence that the velocities of articulators depend on movement direction. For example, it was observed that, on average, lip retraction is faster than lip protrusion [8], that tongue backing is slower than tongue fronting [9–11], that vocal fold adduction is slower than vocal fold abduction [12], and that for men, velum raising is faster than velum lowering [13].

For vertical larynx movements, it is not yet entirely clear whether there are intrinsic velocity differences between larynx lowering and larynx raising. Indirect evidence in this direction is provided by the observation that speakers and untrained singers vary their larynx height with pitch [14–16], and that the maximum speed of pitch change is higher for pitch lowering than for pitch raising [17]. From this it could be concluded that larynx lowering is faster than larynx raising, although a direct relationship between larynx height and pitch, especially in naturally running speech, is not undisputed [18, 19], and is a subject of ongoing research [20, 21]. Further indirect evidence for intrinsic velocity differences in vertical larynx movements is provided by insights into the structure of the biomechanical system. It was found that there are slightly more fast-contracting and fewer slow-contracting fibers in the suprayhyoid muscles than in the infrahyoid muscles [22], which are primarily responsible for larynx raising and for larynx lowering, respectively [23, 24]. This indicates that larynx raising might be intrinsically faster than larynx lowering. Furthermore, it was noted that the biomechanical system includes “not only a great number of muscles but also the often overlooked tracheal pull” [25], which is caused by the mass and the tension of the trachea [26]. The existence of such a permanent downward-directed force could mean that, in contrast to what is indicated by the properties of the extrinsic laryngeal muscles, larynx lowering might be faster than larynx raising. While the structure of the biomechanical system suggests that there are intrinsic velocity differences in the vertical larynx movement, it is so far unclear which part of the biomechanical system dominates this movement, and thus also which direction and size the velocity differences might have.

In general, there are not many studies that measured vertical larynx movement because the measurement is technically demanding and because no standard measurement method has yet been established. Some methods rely on the shape change of the outer throat contour when the larynx moves, and therefore require subjects with a marked thyroid protuberance [18, 27–29]. Another method derives the vertical larynx position from voltages that are measured by means of an electrode array at the height of the glottis [30]. Limitation of this method are that the interpretation of the measured voltages is not straightforward and that it does not yet offer satisfactory repeatability and accuracy. Other methods are based on imaging of the larynx using X-ray cinematography [19, 31] or MRI [24]. X-ray cinematography is often inadequate for the measurement of larynx movements due to the poor contrast of the cartilages, and it is in general hardly used in speech studies anymore because of its ionizing radiation. MRI of the vocal tract is rather expensive, often provides only a limited temporal resolution, and requires subjects to be in an unnatural supine position. An alternative without the limitations of the methods that were mentioned above was introduced recently [21]. There, laryngeal ultrasound was used to infer vertical larynx position from an optical flow analysis in an ultrasound video. This technique was used in the present study since it is easy-to-use, non-invasive, applicable to a wide variety of subjects, and because it provides sufficient temporal resolution.

The goal of the present study was to gain more insights into possible intrinsic velocity differences between larynx raising and larynx lowering by means of a tailored experimental design, where subjects produced alternating raising and lowering movements of the larynx in vowel sequences at specific speaking rates. In this way, the phonetic context was precisely controlled, so that differences between the observed raising and lowering movements could be attributed solely to biomechanical and aerodynamic factors. Learning about intrinsic direction-dependent velocities of articulators cannot only benefit the interpretation of kinematic speech data, but can also help to improve the realism and the quality of articulatory speech synthesis [32–34]. For example, it was found that different intrinsic direction-dependent velocity components of tongue movement can explain and reproduce observed loop patterns in tongue body trajectories [10, 11]. Modeling such more realistic tongue trajectories, as opposed to straight-line paths between articulatory targets, was found to lead to a more natural synthetic speech generated by articulatory synthesis [35, 36]. A better model for the movement of other articulators like the larynx might further improve the realism and the quality of articulatory speech synthesis.

## Materials and methods

### Data acquisition

**Fig 1:**
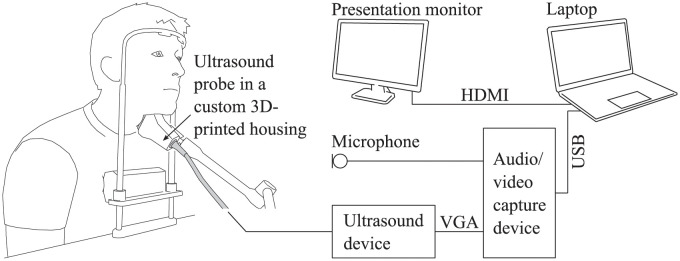
Measurement setup. The subject uttered the sequences that were displayed on the presentation monitor. A frame that served as a rest for the forehead and for the sternum helped the subject maintaining a constant position throughout the experiment. The subject’s vertical larynx movements were recorded by means of laryngeal ultrasound. The resulting ultrasound video was stored on a laptop together with time-synchronous audio.

The vertical larynx movements of 23 native German adults (19 to 48 years old, 12 men and 11 women) without any known speaking or hearing disorders were measured using the setup that is shown in Fig 1. Each subject sat in an upright position and was supported by means of a frame that was attached to a tabletop and served as a rest for the forehead and for the sternum. An ultrasound probe (SonoScape Phased Array 2P1) in a custom 3D-printed housing was placed on the skin in the thyroid lamina region, and fixed in this position by an adjustable and lockable friction arm (Manfrotto 244). The ultrasound video was recorded in B-mode and mostly with a probe pulse frequency of 10 MHz, a scanning depth of 2.8 cm, and a frame rate of 54 Hz. In one third of the measurements, video quality could be improved by changing the probe frequency to 12 MHz or to 15 MHz, or the scanning depth to 3.7 cm. The ultrasound video was forwarded from the ultrasound device (SonoScape S2) to a laptop and recorded there, such that the recording time was not limited by the internal storage of the ultrasound device. The video interface adaptation between the VGA output of the ultrasound device and the USB input of the laptop was achieved using a video capture device (StarTech USB3HDCAP) that also allowed synchronous audio to be added to the ultrasound recording. The sequences to utter were presented visually via an external monitor.

The sequences, which were tailored to elicit pronounced vertical larynx movement, consisted of cyclic transitions between two vowels V1 and V2 of the form V1-V2-V1-…with at least five cycles. The vowel pairs V1-V2 were all 16 combinations of the German rounded vowels /o,u,y,ø/ with the unrounded vowels /i,e,ε,a/, where the rounded vowels were uttered at a low pitch and the unrounded vowels were uttered at a high pitch. Since German rounded vowels are usually produced with lower larynx position than unrounded vowels [37], and since many speakers and untrained singers vary their larynx height with pitch [16], most of these sequences should elicit cyclic changes in the vertical larynx position. The actual pitches were selected by the subject, but should be at least one octave apart. In order to avoid variations in the speaking rate, the subjects were instructed to regulate it according to a metronome. All sequences were uttered both at a slow speaking rate with 500 ms per vowel and at a fast speaking rate with 375 ms per vowel to explore the potential effect of the speaking rate on the vertical larynx movement later on. A subject may still involuntarily emphasize either all V1 or all V2 in a sequence, depending on which vowel the sequence started with. To compensate for this potential issue later on, each subject uttered each sequence twice, once starting with V1 and once with V2. In total, this led to 64 sequences to utter by each subject (16 vowel pairs x 2 speaking rates x 2 start vowels). Three speakers had problems to control their speaking rate or to realize the correct vowel and pitch changes, which resulted in very small or irregular larynx movements. Therefore, the recordings of these three speakers were discarded completely, such that recordings of 10 male speakers and of 10 female speakers were available for the further analysis.

This study was reviewed and approved by the Ethics Committee at the TU Dresden (approval number EK 114032017). From each subject, informed consent was obtained in written form before the experiment.

### Data processing

**Fig 2:**
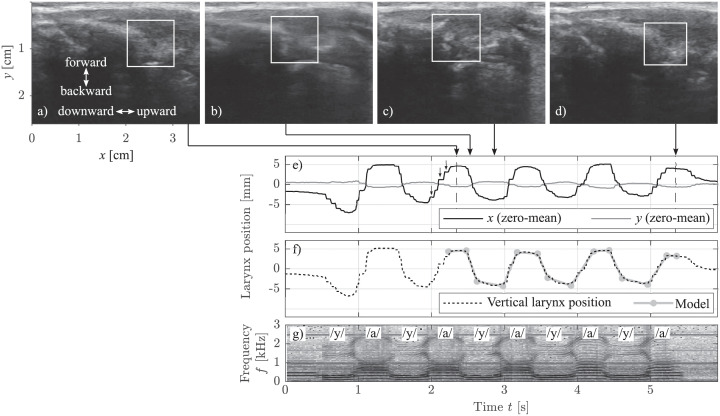
The consecutive data processing steps, illustrated by means of the utterance /y-a-y-…/ of subject M02 at the slow speaking rate, where each /a/ was uttered at a high pitch and is associated with a raised larynx, and each /y/ was uttered at a low pitch and is associated with a lowered larynx. a–d) The tracked region (white rectangle) in the ultrasound image during /a/ after the sequence start, during the transition from /a/ to /y/, during /y/, and during /a/ at the sequence end, respectively. e) The *x*-component (black curve) and the *y*-component (gray curve) of the vertical larynx movement were determined as the center coordinates of the tracked region over time. The artifacts (arrows) result from the adaption of the ultrasound device frame rate to the VGA standard. f) The vertical larynx position (black dashed curve) and the fitted model of straight-line pieces (gray lines) with the optimized endpoint coordinates (gray circles). g) The annotated spectrogram of the time-synchronous audio.

Using the audio data as a time reference, the captured ultrasound video files were segmented into the individual utterances using the video editor VirtualDub2. Each utterance was stored as a series of 8 bit grayscale images with a resolution of 620 × 450 pixels. Using a custom-made software written in Python 3.7, the vertical larynx movement in each utterance was tracked across the ultrasound image sequence using the discriminative correlation filter [38] that is available as the class TrackerCSRT in OpenCV 4.5. For the initialization of the tracking, a rectangular region was selected manually around the first clear structure under the skin in a region of synchronous movement and small deformations. Fig 2a–2d show example video frames with different larynx states during the utterance /y-a-y-…/ of subject M02 at the slow speaking rate. It is well-known that ultrasound videos are generally noisy and of reduced image quality and image clarity, and that the various structures of the larynx are continually moving into and out of the imaging plane during speech [21], which can also be seen here. After a visual validation of the tracking, the *x*-component and the *y*-component of the larynx position (see Fig 2e) were derived as the center coordinates of the tracked region over time. From these curves, only a time interval of three movement cycles, which showed regular movement without artifacts (dashed lines in Fig 2e), was considered for further processing. Since a slight difference between the *x*-axis of the ultrasound image and the vertical movement axis of the larynx was unavoidable, the vertical movement axis of the larynx was determined as the main movement line in the image plane by means of principle component analysis. Then, the *x*-component and the *y*-component of the vertical larynx position were projected onto this line, as described in more detail in a previous publication [13]. The projected signal often contained a linear drift, which was determined by means of linear regression, and subtracted, to yield the vertical larynx position that is shown in Fig 2f. The observed drift, which usually was directed upwards (see *x* in Fig 2e) can be interpreted as both an active compensation for a declining subglottal pressure, and a passive consequence of a decreasing tracheal pull [18, 21, 39, 40].

The vertical larynx position was approximated in the selected time interval by a sequence of straight-line pieces. Each period was represented by four line pieces: one for the raising edge, one for the quasi-stationary phase with raised larynx, one for the falling edge, and one for the quasi-stationary phase with lowered larynx. The endpoint coordinates of the line pieces were initialized semi-automatically and then optimized in such a way that the mean squared error between the curve and its piece-wise linear approximation was minimized (Fig 2f). The optimization was performed with the function fminsearch in Matlab R2021a, which is a derivative-free simplex search method [41]. The absolute slopes of the line pieces for the raising and for the falling edges were taken as approximations of the larynx raising velocities and of the larynx lowering velocities, respectively. This approach based on line pieces was more robust against noise and prevented ambiguities compared to other methods, such as smoothing the curve and then taking the extrema of the first derivative as approximations of the velocities.

**Fig 3:**
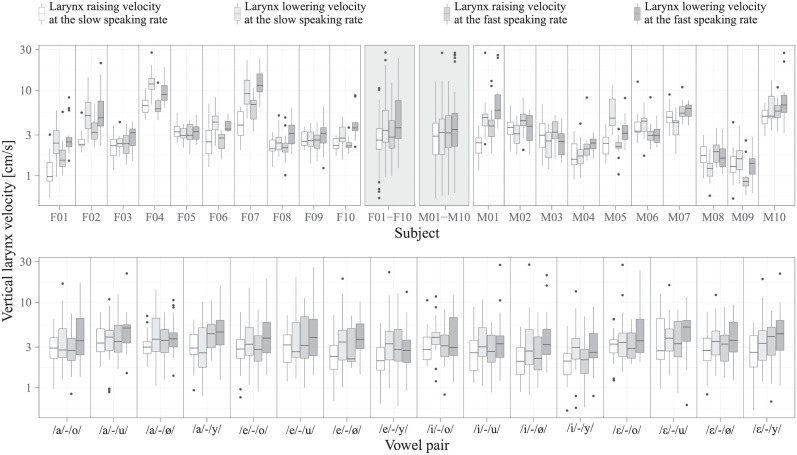
Overview of the data. For each woman F01, F02, …, F10 (top left), for each man M01, M02, …, M10 (top right), and for each vowel pair (bottom), respectively, the first boxplot shows the larynx raising velocities at the slow speaking rate, the second boxplot shows the larynx lowering velocities at the slow speaking rate, the third boxplot shows the larynx raising velocities at the fast speaking rate, and the fourth boxplot shows the larynx lowering velocities at the fast speaking rate, where the brightness decreases from the first to the fourth boxplot. For the visualization, the vertical larynx velocities for each subject were pooled across all vowel pairs, the vertical larynx velocities for each vowel pair were pooled across all subjects. Additionally, in order to highlight sex-dependent effects, the vertical larynx velocities for each sex were pooled across all subjects of that sex and across all vowel pairs (top middle, gray background).

For each combination of subject, vowel pair, and speaking rate, one median larynx raising velocity and one median larynx lowering velocity was calculated across the three movement cycles in the selected time interval and across both vowel pair orders. Only for 2% of the combinations no median value could be calculated since the corresponding data for this combination had to be discarded completely due to invalid tracking or movement artifacts. Fig 3 gives an overview about the resulting data, which is provided in S1 File). The visual and statistical analysis of the data is detailed in the next section. The observed values for the vertical larynx velocity were mainly in the range of 1 cm/s to 10 cm/s, which is consistent with observations by others [42].

### Statistical analysis

The following linear mixed-effects model was fitted to the data in Fig 3 using R 4.2.0, and the lme4 and the lmerTest packages [43, 44]:

$$\begin{array}{cc} \text{log}\;\underset{v_{i,j}}{\underset{︸}{\left(\begin{array}{c} v_{i,j,\text{slow},\text{raising}}\\ v_{i,j,\text{slow},\text{lowering}}\\ v_{i,j,\text{fast},\text{raising}}\\ v_{i,j,\text{fast},\text{lowering}} \end{array}\right)}} & =\underset{X_{i,j}}{\underset{︸}{\left(\begin{array}{cccccc} 1 & \frac{s_{i}}{2} & -\frac{1}{2} & -\frac{1}{2} & -\frac{s_{i}}{4} & \frac{1}{4}\\ 1 & \frac{s_{i}}{2} & -\frac{1}{2} & \frac{1}{2} & \frac{s_{i}}{4} & -\frac{1}{4}\\ 1 & \frac{s_{i}}{2} & \frac{1}{2} & -\frac{1}{2} & -\frac{s_{i}}{4} & -\frac{1}{4}\\ 1 & \frac{s_{i}}{2} & \frac{1}{2} & \frac{1}{2} & \frac{s_{i}}{4} & \frac{1}{4} \end{array}\right)}}\cdot (\begin{array}{c} \beta_{0}\\ \beta_{1}\\ \beta_{2}\\ \beta_{3}\\ \beta_{4}\\ \beta_{5} \end{array})+\underset{Z_{i,j}}{\underset{︸}{\left(\begin{array}{cccc} 1 & 0 & 0 & 1\\ 1 & 0 & 1 & 1\\ 1 & 1 & 0 & 1\\ 1 & 1 & 1 & 1 \end{array}\right)}}\cdot (\begin{array}{c} b_{i,0}\\ b_{i,1}\\ b_{i,2}\\ b_{j} \end{array})+\underset{\epsilon_{i,j}}{\underset{︸}{\left(\begin{array}{c} \epsilon_{i,j,\text{slow},\text{raising}}\\ \epsilon_{i,j,\text{slow},\text{lowering}}\\ \epsilon_{i,j,\text{fast},\text{raising}}\\ \epsilon_{i,j,\text{fast},\text{lowering}} \end{array}\right)}},\\ & \;\;(\begin{array}{c} b_{i,0}\\ b_{i,1}\\ b_{i,2} \end{array})∼N(0,\Psi ),\;b_{j}∼N(0,\sigma_{4}^{2}),\;\epsilon_{i,j}∼N(0,\sigma_{5}^{2}I), \end{array}$$ (1)

where ***v***_*i*,*j*_ were the four vertical larynx velocities (2 speaking rates × 2 movement directions) for the *i*-th subject and the *j*-th vowel pair, ***X***_*i*,*j*_, ***Z***_*i*,*j*_, and ***ϵ***_*i*,*j*_ were the fixed-effects model matrix, the random-effects model matrix, and the residuals, respectively, and *s*_*i*_ was a dummy variable for the sex with *s*_*i*_ = −1 for women and *s*_*i*_ = 1 for men. The corresponding formula for the model specification was

$$\begin{array}{c} \text{log}(v)∼\text{dir}+\text{rate}/\text{dir}+\text{sex}/\text{dir}+(\text{rate}+\text{dir}|\text{subject})+(1|\text{pair}), \end{array}$$

where *pair* stands for vowel pair, *rate* stands for speaking rate, and *dir* stands for movement direction, i.e., for larynx raising or for larynx lowering. In the following, the model is motivated and the model parameters are explained in detail. The model considered a mean larynx velocity *β*_0_ across all subjects, all vowel pairs, both speaking rates and both movement directions. The model furthermore considered the fixed effects *β*_1_, *β*_2_, and *β*_3_ for sex, speaking rate, and movement direction, respectively. Since the objective of the present study was to find out the overall velocity ratio between larynx raising and larynx lowering, and whether it *changes* with the sex or with the speaking rate, the model furthermore considered the interaction effects *β*_4_ and *β*_5_ between sex and movement direction, and between speaking rate and movement direction, respectively. In order to account for the by-subject and the by-vowel-pair variability that is apparent in Fig 3, the model considered a by-subject mean larynx velocity *b*_*i*,0_, a by-subject effect *b*_*i*,1_ of the speaking rate, a by-subject effect *b*_*i*,2_ of the movement direction, and a by-vowel-pair mean larynx velocity *b*_*j*_ as random effects. Thereby, *b*_*i*,0_, *b*_*i*,1_, *b*_*i*,2_ were considered as normally distributed random variables with a mean of zero and a standard deviation of *σ*_1_, *σ*_2_, and *σ*_3_, respectively, with possible correlation coefficients *ρ*_1_, *ρ*_2_, and *ρ*_3_ between *b*_*i*,0_ and *b*_*i*,1_, *b*_*i*,0_ and *b*_*i*,2_, and *b*_*i*,1_ and *b*_*i*,2_, respectively, all of which are the parameters of the symmetric covariance matrix *Ψ* in Eq 1. Likewise, *b*_*j*_ and *ϵ*_*i*,*j*_ were considered as normally distributed random variables with a mean of zero and a standard deviation of *σ*_4_ and *σ*_5_, respectively. For a better interpretability of the fixed effects, the coding scheme of ***X***_*i*,*j*_ was changed from the default dummy-coding (as in ***Z***_*i*,*j*_) to effect-coding. The logarithmic transformation of the vertical larynx velocity was used to meet the model assumptions about homoscedasticity and normality of the residuals, both of which were validated visually. Note that the modeled effects represent velocity *differences* on the logarithmic scale and thus velocity *ratios* on the original scale, which agrees well with the present study, as its aforementioned objective also refers to velocity ratios.

## Results

The estimated fixed-effects parameters are given in Table 1. The effect *β*_1_ of the sex was found to be non-significant, which means that the mean vertical larynx velocity *β*_0_ was about the same for women as for men. The effect *β*_2_ of the speaking rate was found to be significant (*p* = 0.022) and positive, which means that the vertical larynx velocity increased with the speaking rate. The effect of the movement direction was also found to be significant (*p* = 0.003) and estimated as exp(*β*_3_) = 1.26, which means that larynx lowering was on average 26% faster than larynx raising. The interaction effect *β*_4_ between the sex and the movement direction was found to be only slightly above the significance level of 5% (*p* = 0.069) and was therefore also looked at more closely. The meaning of this effect is that the overall velocity ratio between larynx lowering and larynx raising was exp(*β*_3_ − 0.5 ⋅ *β*_4_) = 1.44 (*p* = 0.002) for women, and exp(*β*_3_ + 0.5 ⋅ *β*_4_) = 1.11 (*p* = 0.301) for men, where the *p*-values were derived under the null hypothesis that the velocity ratio is smaller or equal to one. In other words, larynx lowering was on average 44% faster than larynx raising for women, while there was no such pronounced difference for men. The interaction effect between the speaking rate and the movement direction was found to be non-significant, which means that, although the speaking rate had an effect on the mean vertical larynx velocity, it had no such effect on the overall velocity ratio between larynx raising and larynx lowering. In a graphical way, these results become particularly evident through the grouping of the vertical larynx velocities with respect to sex (F01–F10 and M01–M10 in Fig 3).

**Table 1:** Estimated fixed-effects parameters. The parameters were determined by fitting the linear mixed-effects model in Eq 1 to the data in Fig 3 and are given in terms of the estimate and the standard error on the logarithmic scale, the degrees of freedom (DF), the *t*-value, and the *p*-value, where *p*-values below a significance level of 5% are written in bold.

Parameter	Estimate	Standard error	DF	*t*-value	*p*-value
*β*_0_	1.151	0.113	19.7	10.2	<**0.001**
*β*_1_	-0.157	0.221	18.0	-0.7	0.486
*β*_2_	0.103	0.041	19.2	2.5	**0.022**
*β*_3_	0.233	0.070	18.1	3.4	**0.003**
*β*_4_	-0.266	0.137	18.1	-1.9	0.069
*β*_5_	-0.030	0.043	1179.6	-0.7	0.484

The random-effects parameters were *σ*_1_ = 0.24, *σ*_2_ = 0.03, *σ*_3_ = 0.09, *σ*_4_ = 0.01, *σ*_5_ = 0.14, *ρ*_1_ = 0.03, *ρ*_2_ = 0.31, and *ρ*_3_ = 0.12, and the variance that was explained by the fixed and by the random effects was *R*^2^ = 0.68.

## Discussion and conclusions

**Fig 4:**
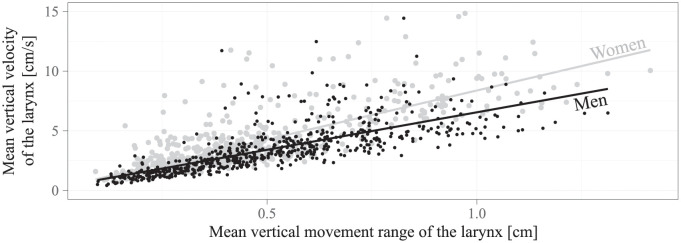
Sex-dependent linear relationship between the mean vertical movement range of the larynx and the mean vertical velocity of the larynx. Each point represents the value pair for one specific combination of subject, vowel pair, and speaking rate. The linear relationships that were found for the women (*ρ* = 0.778, *p* < 0.001) and for the men (*ρ* = 0.591, *p* < 0.001) are displayed in terms of the regression lines. For visualization purposes, five points per sex in the range from 15 cm/s to 30 cm/s are not displayed here. The intercept and the slope of the regression lines were 0.13 cm/s and 6.85 Hz, respectively, for men, and -0.04 cm/s and 8.82 Hz, respectively, for women.

The main result of the present study was that larynx lowering was on average 26% faster than larynx raising. A possible explanation for this is that both the mass of the larynx (and the connected structures) and the tension of the trachea introduce permanent downward-directed forces into the dynamics of the larynx. Another finding was that both the larynx raising velocity and the larynx lowering velocity increased by the same factor with the speaking rate. On the one hand, this shows the plausible influence of the active control by the nervous system on the absolute vertical larynx velocity. On the other hand, since both the larynx lowering velocity and the larynx raising velocity increased by the same factor, their ratio seems to be not under active control. Apart from this, the aerodynamic conditions were considered to be of minor importance, since the vowel sequences that were used here do not require a critical constriction in the vocal tract so that the airflow can hardly exert any considerable force on the articulators. This suggests the properties of the biomechanical system as the main cause for the observed velocity ratio, and consequently a sex-dependent difference in the biomechanical properties as the main cause for the observed sex-dependent difference in the velocity ratio. In order to elaborate on this, the mean vertical movement range was calculated as the mean travel distance of the larynx during the rising and the falling edges that are illustrated in Fig 2f, and plotted against the vertical velocity of the larynx, as shown in Fig 4. A linear relationship was found for both sexes (*p* < 0.001), which points towards the involvement of a spring-mass system with a linear mass-normalized stiffness [4], where a greater slope points towards a greater stiffness. A biomechanical structure that could introduce a greater stiffness in women, i.e., a greater necessary force for the *same absolute elongation*, which in the present study was indeed indicated by the non-significant difference in the vertical movement range of the larynx between both sexes (*p* = 0.631), is the trachea [45], possibly due to its shorter resting length compared to men. This suggests that there may be a larger downward-directed tracheal restoring force in women, which could both increase the larynx lowering velocity and decrease the larynx raising velocity, and thereby explain why larynx lowering was faster and larynx raising was slower compared to men.

The interpretation of the this from an evolutionary point of view could shed light on *why* the biomechanical properties may have developed in this way. In general, there is consensus that the evolution of the larynx has been determined primarily by the basic functions of airway-opening and airway-closing, rather than the phytogenetically younger function of phonation. Since both airway-opening and airway-closing are essential, namely for respiration and for the prevention of aspiration, respectively, there are different opinions about which of these basic functions had a greater impact on the evolution of the larynx. Some think that it was the airway-closing, which allowed for the independent and more efficient use of the forelimbs through the so-called air-trapping [46]. Air-trapping, which is also known as effort closure, is a technique to stabilize the thorax by preventing the passage of air into the lungs [47]. Others think that it was the airway-opening, which allowed for quick and increased air intake for sprinting and endurance running [48]. This view is supported the spring-like recoiling structures in the larynx [49] that seem to favour airway-opening over airway-closing. What links this discussion to the present study is that the laryngeal axis of constriction, i.e., airway-opening and airway-closing, and the laryngeal axis of height, i.e., vertical larynx movement, have been noted to be interdependent [42]. In particular, it has been noted that “lifting the larynx up is part of a recruitment chain that entails other parallel actions, namely, engaging the aryepiglottic sphincter”, and that“the lowered-larynx setting, as the polar opposite of constriction, predisposes opening of the airway” [50]. From this, it can be deduced that larynx raising and larynx lowering are to some extent associated with airway-closing and airway-opening, respectively. The finding of the present study that larynx lowering was faster than larynx raising therefore provides more evidence in the direction of airway-opening as the main driving force in the evolution of the larynx. The finding that the velocity ratio is more balanced in men than in women could indicate that the airway-closing, which allows for an increase in upper-body strength, has nevertheless been a somewhat competing evolutionary force in men.

**Fig 5:**
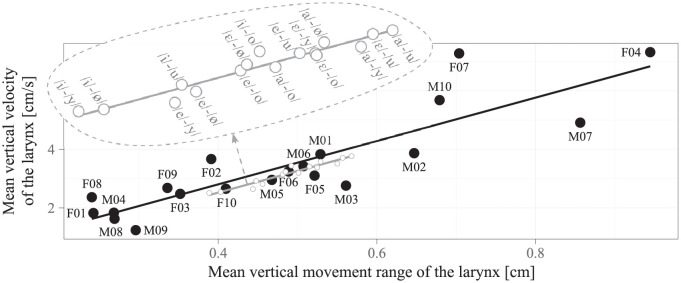
Linear relationships between the mean vertical movement range of the larynx and the mean vertical velocity of the larynx across the subjects and across the vowel pairs. Each black point represents the value pair for one specific subject, for which the values were pooled across both the vowel pairs and the speaking rates, and each gray point represents the value pair for one specific vowel pair, for which the values were pooled across both the subjects and the speaking rates. The linear relationships that were found for the subjects (*ρ* = 0.812, *p* < 0.001) and for the vowel pairs (*ρ* = 0.936, *p* < 0.001) are displayed in terms of the regression lines. The intercept and the slope of the regression lines were -0.15 cm/s and 7.40 Hz, respectively, for subjects, and -0.43 cm/s and 7.38 Hz, respectively, for vowel pairs.

Another finding of the present study were the considerable by-subject variations and the somewhat smaller by-vowel-pair variations in the vertical movement velocity of the larynx, which are evident in Fig 2. For a closer examination of possible causes for the subject-dependent variations, the by-subject mean vertical movement velocity of the larynx, mean velocity ratio between larynx lowering and larynx raising, and mean velocity ratio between vertical larynx movements at the fast and at the slow speaking rate were each correlated with age, body weight, body height, and mean vertical movement range of the larynx. Analogously, for a closer examination of a possible cause for the vowel-pair-dependent variation, the by-vowel-pair mean vertical movement velocity of the larynx was correlated with the mean vertical movement range of the larynx. Significant correlation coefficients of *ρ* = 0.812 (*p* < 0.001) and *ρ* = 0.936 (*p* < 0.001) were found only between the mean vertical velocity of the larynx and the mean vertical movement range of the larynx, as shown in Fig 5. This means that the mean vertical velocity of the larynx varied with the mean vertical movement range of the larynx, which in turn was a function of the subject and of the vowel pair, respectively, as also observed elsewhere [37]. It furthermore means that the cause for the other by-subject variations, as well as the possible influences of age, body weight, and body height, could not be identified in such a clear way. Interestingly, significant linear relationships between the mean vertical movement range of the larynx and the mean vertical velocity of the larynx were observed across all subjects (see Fig 5), within both sexes (see Fig 4), and, as an additional analysis of the data in Fig 4 revealed, also within each subject. Following the discussion further above, these linear relationships indicate the involvement of a spring-mass system with a sex-dependent and also subject-dependent linear mass-normalized stiffness. The sex-dependent stiffness may explain why larynx lowering was faster and larynx raising was slower for women compared to men. There was however no correlation between the subject-dependent stiffness and the subject-dependent velocity ratio between larynx lowering and larynx raising, which thus seems to have additional causes beyond the scope of the present study. Apart from that, there is also another interesting, rather kinematical interpretation of the linear relationship between the mean vertical movement range of the larynx and the the mean vertical velocity of the larynx. The relatively small intercepts close to zero that are illustrated in Figs 4 and 5 point towards a direct proportionality between both quantities. This would mean that their ratio, i.e., the time needed to approach a new target in the vertical larynx position, is more or less constant and thus independent of the associated movement range.

In summary, the present study indicates that the larynx lowering is faster than the larynx raising, that this difference is not as pronounced in men as in women, and that all of this could be explained by specific properties of the biomechanical system, which are to some extent sex-dependent and subject-dependent. The results could help to better understand the complex nature of speech kinematics, with possible implications on articulatory speech synthesis and on interpretation of articulator trajectories. The observed velocity differences between larynx lowering and larynx raising characterize the healthy state of the larynx and connected structures such as the extrinsic laryngeal muscles. Possible clinical implications of this, e.g., in the context of muscle tension dysphonia [51], were not the focus here. Our future work will mainly focus on the potential benefit of including these velocity differences in articulatory speech synthesis.

## Supporting information

S1 File Raw vertical larynx velocities. For all 1280 sequences (20 subjects × 16 vowel pairs × 2 vowel pair orders × 2 speaking rates), there are three measurements according to the three movement cycles in the selected time interval. Each measurement corresponds to one velocity value for larynx lowering, and one for larynx raising, both of which are given in cm/s. The vowel pair order is the index of the start vowel, e.g., for the vowel pair /i-o/, vowel pair order one means that the sequence started with /i/ while vowel pair order two means that the sequence started with /o/. SAMPA symbols are used for all vowels. Furthermore, the subject-specific age in years, body height in cm, and body weight in kg are given, as well as the sequence-specific mean movement range in cm.
(CSV)

S2 File Author-generated code that underpins the findings of the present study. Only S1 File is needed as an input.
(R) Click here for additional data file.
